# Knowledge, attitudes, and practices of Lebanese physicians on the screening of pediatric dyslipidemias: a KAP cross-sectional study

**DOI:** 10.3389/fendo.2026.1900463

**Published:** 2026-09-09

**Authors:** Marie-Hélène Gannagé-Yared, Christy El Habre, Bachir Atallah, Michel Bou Absi, Nada El Ghorayeb, Maissa Safieddine

**Affiliations:** 1 Department of Endocrinology, Hôtel-Dieu de France hospital, Faculty of Medicine, Saint-Joseph University, Beirut, Lebanon; 2 Clinical Research Center, Faculty of Medicine, Saint-Joseph University, Beirut, Lebanon

**Keywords:** adolescents, attitudes, children, dyslipidemia, knowledge, Lebanon, physicians, practices

## Abstract

**Introduction:**

Pediatric dyslipidemia is common worldwide, especially in Lebanon, and is associated with increased long-term cardiovascular risk. However, international screening recommendations are still controversial. In Lebanon, no study has assessed physicians’ knowledge, attitudes, and practices (KAPs) about this screening.

**Methods:**

A cross-sectional online questionnaire-based study was conducted among pediatricians, family medicine physicians, and endocrinologists practicing in Lebanon. The questionnaire assessed knowledge, attitudes, screening practices, and perceived barriers regarding pediatric dyslipidemia. Associations between variables were examined using univariate and multivariable analyses.

**Results:**

A total of 245 physicians participated in the study. Performance on the nine selected knowledge items was low (mean score of 53.2 ± 22.1 out of 100, median 55.6), with gaps mainly related to the prevalence of pediatric dyslipidemia in Lebanon and familiarity with international recommendations. Attitudes toward screening were generally favorable (mean score of 77.8 ± 14.2 out of 100, median 80), although 56.3% of participants reported concerns about family anxiety. Screening practices were heterogeneous and primarily relied on targeted screening when risk factors were present. Screening in adolescents was performed (always or most of the time) by 40.3% of endocrinologists, 32.6% of family medicine physicians, and 19.3% of pediatricians, with laboratory test costs and parental consent being the main obstacles (49.8% and 33.9%, respectively). The score based on the nine selected knowledge items showed a weak positive correlation with the attitude score (Spearman’s ρ = 0.135, p = 0.035). Attitude scores differed significantly across reported screening-frequency categories (p<0.001). In multivariable analysis, female sex, family medicine practice, and practice in Beirut/Mount Lebanon (BML) were independently associated with higher score on the nine selected knowledge items (p=0.015, p<0.001, and p=0.005, respectively).

**Conclusion:**

Despite favorable attitudes, insufficient knowledge and contextual barriers limit the systematic implementation of pediatric dyslipidemia screening in Lebanon. Actions that combine training, harmonization of recommendations, and improved access to screening could strengthen pediatric cardiovascular prevention.

## Introduction

Cardiovascular diseases are the leading cause of death worldwide and represent a major public health burden (1). Although traditionally considered diseases of adulthood, accumulating epidemiological and pathophysiological evidence has shown that atherosclerosis begins early in life and that lipid profiles during childhood and adolescence are associated with cardiovascular events later in life (2–4).

Available data highlighted a concerning prevalence of lipid abnormalities in children. In the United States (US), nearly one in five children had dyslipidemia (5). In Lebanon, two studies reported significant proportions of children with elevated levels of non-HDL cholesterol (9% and 19%, respectively) and high triglycerides (26% and 31.8%, respectively) (6, 7). These abnormalities were also observed in children without obvious clinical risk factors (6, 7), which strengthens the argument for universal screening.

Despite these findings, international recommendations regarding screening for pediatric dyslipidemia remain heterogeneous. The National Heart, Lung, and Blood Institute, supported by the American Academy of Pediatrics (AAP), recommends universal screening at ages 9–11 and 17–21 years, as well as targeted screening starting at age 2 for children with risk factors (8). Conversely, the U.S. Preventive Services Task Force (USPSTF) considers current data insufficient to assess the risk-benefit ratio of systematic screening in asymptomatic children and adolescents (9). This lack of consensus is likely to influence clinical practices and generate variability in the application of screening strategies (10–12).

Despite the high prevalence of pediatric dyslipidemia, lipid screening in children remains insufficient in many countries. Studies report that only 3.4% to 16% of children underwent lipid screening between 1995 and 2012 (13–15). Following the AAP recommendations, however, an increase in this practice was observed, with nearly one-third of young people aged 16 to 21 years having already undergone lipid screening (5). However, a recent decline in cholesterol screening has been reported following the COVID-19 pandemic, and after the USPSTF updated its guidelines, which adopted a more cautious position regarding universal screening (16).

Several factors, in addition to heterogeneous guidelines, contribute to this low screening rate, including insufficient knowledge and attitudes among physicians regarding current recommendations. A survey conducted in the US between 2013 and 2014 has shown that universal screening was not routinely performed and that only 26% of pediatricians reported being familiar with current pediatric lipid screening recommendations (17). Conversely, another study conducted after the implementation of training and awareness programs for pediatricians regarding the recommendations demonstrated a significant increase in the screening rate, rising from 8.9% to 50% within 12 months (18).

To date, no study has evaluated the knowledge, attitudes, and practices of Lebanese physicians regarding pediatric dyslipidemia screening. Understanding these dimensions is essential to identify existing gaps, explore determinants of screening practices, and guide context-specific strategies adapted to the Lebanese healthcare setting. Therefore, this study aimed to assess the knowledge, attitudes, and practices of pediatricians, family medicine physicians, and endocrinologists practicing in Lebanon regarding pediatric dyslipidemia, and to identify the factors associated with these dimensions.

## Materials and methods

This cross-sectional KAP (Knowledge, Attitudes, and Practices) study was conducted between October 2025 and January 2026 among pediatricians, endocrinologists, and family medicine physicians practicing in Lebanon. Eligible participants included physicians currently practicing in university hospitals or in private clinics. Data were collected using a self-administered electronic questionnaire distributed in English and French through professional medical societies (WhatsApp groups and recruitment at the annual meeting of the societies)using a voluntary, non-probability, society-based sampling.

### The KAP questionnaire

The questionnaire was designed according to the KAP survey model (19). It was initially designed in French based on international pediatric dyslipidemia screening recommendations and the existing literature. It was subsequently reviewed by three senior physicians with expertise in pediatrics, endocrinology, and family medicine to assess content relevance, clarity, and clinical appropriateness. Revisions were incorporated following expert feedback. To accommodate physicians who preferred to respond in English, the finalized French version was translated into English and then translated back into French to ensure semantic equivalence between the two versions. The final bilingual questionnaire was reviewed before dissemination.

The questionnaire comprised five sections: (1) socio-demographic and occupational characteristics; (2) familiarity with international recommendations; (3) knowledge regarding pediatric dyslipidemia; (4) attitudes toward pediatric dyslipidemia screening, and (5) clinical practices, including screening frequency, prescribing criteria, and perceived barriers.

### Performance on selected screening-related knowledge items and attitude scores

A screening-related knowledge score was calculated from nine selected questionnaire items addressing pediatric dyslipidemia epidemiology and screening recommendations. It was adapted from a previous study (10). The score was developed for the present study and was not derived from a standardized or externally validated knowledge scale. One point was awarded for each correct answer, and the total score was converted to a 100-point scale.

Attitude score was assessed using Likert-scale items ranging from “strongly disagree” to “strongly agree”. The total attitude score was also converted to a 100-point scale.

For both knowledge and attitude scores, higher scores indicate better knowledge and more favorable attitudes. The rationale, correct answers, and supporting references for the nine pediatric lipid-screening knowledge items are presented as a **Supplementary Table 1**.

### Statistical analysis

A descriptive analysis was conducted to characterize the study population. The distribution of continuous variables was assessed using the Kolmogorov-Smirnov test. Categorical variables were presented as frequencies and percentages, while continuous variables were expressed as means with standard deviations and medians with interquartile ranges, as appropriate. Internal consistency was assessed using Cronbach’s alpha coefficient. The Kaiser–Meyer–Olkin (KMO) measure of sampling adequacy and Bartlett’s test of sphericity were used to evaluate the suitability of the data for factor analysis.

Bivariate analyses were performed to identify factors associated with the knowledge and the attitude score. Associations between categorical variables were assessed using Pearson’s chi-square test or Fisher’s exact test, as appropriate. The Mann-Whitney U test was used to compare continuous or ordinal variables between two independent groups. In comparison, the Kruskal-Wallis test was used to compare more than two independent groups. Spearman’s correlation coefficient was used to assess correlations between continuous scores. A multiple linear regression model was performed to identify factors independently associated with the knowledge score. Covariates were selected *a priori* based on their clinical relevance and potential confounding effect rather than solely on statistical significance in the bivariable analysis. Regression assumptions and multicollinearity were assessed before interpreting the model.

Statistical significance was set at p < 0.05 for all analyses. The responses obtained via Google Forms were exported to Microsoft Excel and then analyzed using IBM SPSS Statistics, version 26.

### Ethical considerations

Participation was voluntary, as evidenced by the completion of the questionnaire. The data collected was anonymous and used exclusively for scientific research purposes. The study was approved by the Hotel Dieu de France (HDF) hospital Ethics Committee (CEHDF2726).

## Results

### Response rate

The questionnaire was distributed to active endocrinologists, family medicine physicians, and pediatricians through the relevant professional societies and through direct recruitment at national medical congresses. Among active endocrinologists, 77 of the 187 surveyed physicians completed the questionnaire, yielding a response rate of 41.2%. Among active family medicine physicians, 49 of the 140 surveyed physicians responded, corresponding to a response rate of 35.0%. Among active pediatricians, 119 of the 292 surveyed physicians completed the questionnaire, yielding a response rate of 39.6%.

### Sociodemographic characteristics of participants

The sociodemographic characteristics of the participants are presented in **Table 1**. A total of 245 physicians completed the questionnaire, including 119 pediatricians (48.6%), 77 endocrinologists (31.4%), and 49 family medicine physicians (20.0%). Most respondents were female (n=157; 64.1%) and practiced in a university setting (n=198; 80.8%), whereas 47 (19.2%) practiced in a non-university setting. Regarding geographic distribution, the majority practiced in Beirut/Mount Lebanon (BML) (n=202; 82.4%), while 43 (17.6%) practiced in other Lebanese regions. Finally, 45% had been practicing for more than 20 years.

**Table 1:** General characteristics of the participants.

Sociodemographic characteristics of participants		n (%)
Gender	Male	88 (35.9%)
	Female	157 (64.1%)
Age (in years)	Mean ± SD	47.6 ± 13.3
Specialty	Pediatric	119 (48.6%)
	Endocrinology	77 (31.4%)
	Family Medicine	49 (20.0%)
Years of clinical practice (post-residency)	< 5 years	57 (23.3%)
	5–10 years	23 (9.4%)
	11–20 years	55 (22.4%)
	> 20 years	110 (44.9%)
Type of practice	Non-University	47 (19.2%)
	University	198 (80.8%)
Region of practice	Beirut/Mount Lebanon	202 (82.4%)
	Others	43 (17.6%)

### Psychometric properties of the questionnaire

The score based on the nine selected items demonstrated moderate internal consistency (Cronbach’s alpha = 0.617). Sampling adequacy for factor analysis was limited (KMO = 0.576), although Bartlett’s test of sphericity was significant (p < 0.001).

The attitude scale included six items. This score demonstrated acceptable to moderate internal consistency (Cronbach’s alpha = 0.691). Sampling adequacy was good (KMO = 0.775), and Bartlett’s test of sphericity was significant (p < 0.001).

### Knowledge of pediatric dyslipidemia

#### Physicians’ knowledge of AAP and USPSTF guidelines

Familiarity with pediatric dyslipidemia screening guidelines was moderate. Regarding the AAP guidelines, 33.5% of physicians reported being familiar, whereas 40.8% reported limited or no familiarity. Similarly, 38.4% reported being familiar or very familiar with the USPSTF guidelines, while 41.6% reported limited or no familiarity.

#### Score based on the nine selected knowledge items

Response to the knowledge items is presented in **Table 2**. Overall, physicians demonstrated an insufficient level of knowledge, with a mean composite score of 53.2 ± 22.1 over 100, a median of 55.6, and scores ranging from 0 to 100.

**Table 2:** Knowledge assessment of the total population and by medical specialty (the correct answer was provided at the end of the question).

Knowledge-related questions		Total (N = 245)	Pediatricians (N = 119)	Endocrinologists (N = 77)	Family medicine physicians (N = 49)
What is the estimated prevalence of Familial Hypercholesterolemia (FH) in Lebanon? 1/100 or 1/200		115 (46.9%)	44 (37.0%)	41 (53.2%)	30 (61.2%)
What is the approximate prevalence of elevated triglycerides in Lebanese children aged 8 to 18 years? 0.26		42 (17.1%)	13 (10.9%)	22 (28.6%)	7 (14.3%)
At what age do the American Academy of Pediatrics (AAP) and the NHLBI recommend systemic screening for dyslipidemias? 9–11 years		125 (51.0%)	53 (44.5%)	38 (49.4%)	34 (69.4%)
At what age do the American Academy of Pediatrics (AAP) and the NHLBI recommend systemic screening for dyslipidemias? 17–21 years		41 (16.7%)	12 (10.1%)	11 (14.3%)	18 (36.7%)
According to current recommendations (AAP and NHLBI), which of the following are considered risk factors justifying early lipid screening in children?	Family history of premature cardiovascular disease	192 (78.4%)	85 (71.4%)	64 (83.1%)	43 (87.8%)
	Obesity	199 (81.2%)	101 (84.9%)	55 (71.4%)	43 (87.8%)
	Hypertension	130 (53.1%)	63 (52.9%)	35 (45.5%)	32 (65.3%)
	Diabetes	144 (58.8%)	66 (55.5%)	45 (58.4%)	33 (67.3%)
According to NHLBI and AAP recommendations, which of the following values are considered abnormal in a child? LDL-C ≥ 130 mg/dL and TG ≥ 100 mg/dL (in a child < 10 years)		184 (75.1%)	89 (74.8%)	56 (72.7%)	39 (79.6%)

In bivariate analyses, female physicians had significantly higher scores than male physicians [mean ± SD: 56.1 ± 21.9 vs. 47.9 ± 21.6, median (IQR): 55.6 (44.4–66.7) vs 44.4 (33.3–66.7), p = 0.004]. Score also differed significantly by specialty (p = 0.001). Family medicine physicians had the highest mean scores [mean ± SD: 63.3 ± 21.8; median (IQR): 66.7 (44.4–77.8)], followed by endocrinologists [mean ± SD: 53.0 ± 23.9; median (IQR): 55.6 (33.3–66.7)] and pediatricians [mean ± SD: 49.1 ± 19.8; median (IQR): 55.6 (33.3–66.7)]. Physicians practicing in BML had significantly higher scores than those practicing in other Lebanese regions [55.7 ± 21.1 vs. 41.1 ± 22.9, median (IQR): 55.6 (44.4–66.7) vs. 33.3 (22.2–55.6); p < 0.001] (**Table 3**).

**Table 3:** Factors associated with the score based on the nine selected knowledge items on pediatric dyslipidemias.

Studied factors		Knowledge score		p-value
		Mean ± SD	Median [IQR]	
Gender	Men	47.9 ± 21.6	44.4 [33.3 - 66.7]	**0.004**
	Women	56.1 ± 21.9	55.6 [44.4 - 66.7]	
Age	Less than 45 years	53.5 ± 23.2	55.6 [33.3 - 66.7]	0.847
	45 years and above	52.9 ± 21.3	55.6 [33.3 - 66.7]	
Specialty	Pediatrics	49.1 ± 19.8	55.6 [33.3 - 66.7]	**0.001**
	Endocrinology	53.0 ± 23.9	55.6 [33.3 - 66.7]	
	Family Medicine	63.3 ± 21.8	66.7 [44.4 - 77.8]	
Years of clinical practice (post-residency)	< 5 years	51.3 ± 21.9	55.6 [33.3 - 66.7]	0.113
	5–10 years	47.8 ± 27.7	44.4 [33.3 - 66.7]	
	11–20 years	58.8 ± 19.6	55.6 [44.4 - 77.8]	
	> 20 years	52.4 ± 21.9	55.6 [33.3 - 66.7]	
Type of practice	Non-University	49.2 ± 20.3	55.6 [33.3 - 66.7]	0.162
	University	54.1 ± 22.5	55.6 [33.3 - 66.7]	
Region of practice	Beirut/Mount Lebanon	55.7 ± 21.1	55.6 [44.4 - 66.7]	**<0.001**
	Others	41.1 ± 22.9	33.3 [22.2 - 55.6]	

Significant p values are shown in bold.

The fully adjusted multiple linear regression model was statistically significant, F(9, 235) = 4.73, p <0.001, and explained 15.3% of the variance in the score based on the nine selected knowledge items, with an adjusted R^2^ of 0.121. Female physicians had, on average, a 7.34-point higher score on the nine selected knowledge items than male physicians (95% CI: 1.42 to 13.25; p = 0.015). Family medicine physicians had a 13.22-point higher score than pediatricians (95% CI: 5.88 to 20.55; p <0.001), and physicians practicing in BML had a 10.84-point higher score than those practicing in other regions (95% CI: 3.32 to 18.35; p = 0.005). Age, years of clinical practice, university-based practice, and endocrinology specialty were not independently associated with the score based on the nine selected knowledge items. No substantial multicollinearity was detected. The adjusted generalized variance inflation factors ranged from 1.04 to 2.11 (**Table 4**).

**Table 4:** Multivariable linear regression analysis of factors associated with the score based on the nine selected knowledge items.

Variable	Adjusted B	95% CI	p-value
Region of practice			
Beirut/Mount Lebanon vs Others	10.84	3.32 to 18.35	0.005
Type of practice			
University vs Non-University	0.07	−6.91 to 7.05	0.984
Specialty			
Endocrinology vs Pediatrics	5.48	−0.68 to 11.65	0.081
Family Medicine vs Pediatrics	13.22	5.88 to 20.55	<0.001
Years of clinical practice			
5–10 years vs <5 years	-4.66	−14.89 to 5.57	0.371
11–20 years vs <5 years	9.39	−0.29 to 19.06	0.057
>20 years vs <5 years	9.90	−3.19 to 22.99	0.138
Gender			
Female vs Male	7.34	1.42 to 13.25	0.015
Age			
≥45 years vs <45 years	-5.37	−16.56 to 5.81	0.345

Model statistics: N = 245; R^2^ = 0.153; adjusted R^2^ = 0.121; F(9, 235) = 4.73; p < 0.001.

Adjusted B represents the adjusted mean difference in knowledge score. Reference categories: Others for region, Non-University for type of practice, Pediatrics for specialty, <5 years for years of clinical practice, Male for gender, and age <45 years.

### Attitudes toward screening

Responses to attitude-related items are presented overall and by specialty in **Table 5**.

**Table 5:** Physicians’ attitudes toward pediatric dyslipidemia.

Attitudes-related questions		Total	Specialty		
			Pediatrics	Endocrinology	Family medicine
Pediatric dyslipidemias represent an important public health problem	Strongly disagree	12 (4.9%)	5 (4.2%)	6 (7.8%)	1 (2.0%)
	Disagree	6 (2.4%)	4 (3.4%)	1 (1.3%)	1 (2.0%)
	Neutral	23 (9.4%)	13 (10.9%)	3 (3.9%)	7 (14.3%)
	Agree	103 (42.0%)	51 (42.9%)	33 (42.9%)	19 (38.8%)
	Strongly agree	101 (41.2%)	46 (38.7%)	34 (44.2%)	21 (42.9%)
Early screening allows the prevention of future cardiovascular diseases.	Strongly disagree	18 (7.3%)	11 (9.2%)	5 (6.5%)	2 (4.1%)
	Disagree	5 (2.0%)	3 (2.5%)	2 (2.6%)	0 (0.0%)
	Neutral	8 (3.3%)	3 (2.5%)	3 (3.9%)	2 (4.1%)
	Agree	60 (24.5%)	29 (24.4%)	18 (23.4%)	13 (26.5%)
	Strongly agree	154 (62.9%)	73 (61.3%)	49 (63.6%)	32 (65.3%)
I feel competent to interpret a lipid panel in a child	Strongly disagree	15 (6.1%)	5 (4.2%)	7 (9.1%)	3 (6.1%)
	Disagree	30 (12.2%)	11 (9.2%)	13 (16.9%)	6 (12.2%)
	Neutral	56 (22.9%)	27 (22.7%)	15 (19.5%)	14 (28.6%)
	Agree	103 (42.0%)	56 (47.1%)	27 (35.1%)	20 (40.8%)
	Strongly agree	41 (16.7%)	20 (16.8%)	15 (19.5%)	6 (12.2%)
Pediatric lipid screening makes families anxious	Strongly disagree	19 (7.8%)	10 (8.4%)	4 (5.2%)	5 (10.2%)
	Disagree	40 (16.3%)	27 (22.7%)	8 (10.4%)	5 (10.2%)
	Neutral	48 (19.6%)	20 (16.8%)	16 (20.8%)	12 (24.5%)
	Agree	89 (36.3%)	42 (35.3%)	32 (41.6%)	15 (30.6%)
	Strongly agree	49 (20.0%)	20 (16.8%)	17 (22.1%)	12 (24.5%)
Pediatric lipid screening can be a tool to improve adherence to lifestyle changes	Strongly disagree	17 (6.9%)	9 (7.6%)	4 (5.2%)	4 (8.2%)
	Disagree	5 (2.0%)	1 (0.8%)	2 (2.6%)	2 (4.1%)
	Neutral	11 (4.5%)	6 (5.0%)	2 (2.6%)	3 (6.1%)
	Agree	101 (41.2%)	51 (42.9%)	35 (45.5%)	15 (30.6%)
	Strongly agree	111 (45.3%)	52 (43.7%)	34 (44.2%)	25 (51.0%)
All children should have at least one cholesterol test	Strongly disagree	15 (6.1%)	5 (4.2%)	2 (2.6%)	8 (16.3%)
	Disagree	32 (13.1%)	13 (10.9%)	11 (14.3%)	8 (16.3%)
	Neutral	32 (13.1%)	11 (9.2%)	19 (24.7%)	2 (4.1%)
	Agree	78 (31.8%)	44 (37.0%)	22 (28.6%)	12 (24.5%)
	Strongly agree	88 (35.9%)	46 (38.7%)	23 (29.9%)	19 (38.8%)

The mean attitude score was 77.8 ± 14.2 out of 100 with a median of 80.0. Attitude scores were broadly comparable across participant subgroups, with no statistically significant differences according to gender (p = 0.849), age (p = 0.663), specialty (p = 0.984), years of clinical practice (p = 0.078), type of practice (p = 0.208), or region of practice (p = 0.127) (**Table 6**).

**Table 6:** Factors associated with the score of attitudes towards pediatric dyslipidemia.

Studies factors		Attitude score		p-value
		Mean ± SD	Median [IQR]	
Gender	Men	77.35 ± 16.00	78.4 [70.0 - 90.0]	0.849
	Women	78.13 ± 13.12	80.0 [73.3 - 86.7]	
Age	Less than 45 years	78.88 ± 11.50	80.0 [73.3 - 86.7]	0.663
	45 years and above	77.05 ± 15.97	80.0 [70.0 - 90.0]	
Specialty	Pediatrics	77.87 ± 14.26	80.0 [70.0 - 90.0]	0.984
	Endocrinology	78.09 ± 13.97	80.0 [70.0 - 86.7]	
	Family Medicine	77.41 ± 14.68	80.0 [73.3 - 86.7]	
Years of clinical practice (post-residency)	< 5 years	76.08 ± 13.17	76.7 [70.0 - 86.7]	0.078
	5–10 years	84.06 ± 7.38	83.3 [80.0 - 90.0]	
	11–20 years	77.63 ± 11.88	76.7 [73.3 - 83.3]	
	> 20 years	77.58 ± 16.47	80.0 [70.0 - 90.0]	
Type of practice	Non-University	76.10 ± 15.36	76.7 [70.0 - 83.3]	0.208
	University	78.26 ± 13.91	80.0 [73.3 - 90.0]	
Region of practice	Beirut/Mount Lebanon	75.81 ± 12.44	76.7 [70.0 - 83.3]	0.127
	Others	78.28 ± 14.53	80.0 [73.3 - 90.0]	

### Clinical screening practices

Screening practices are presented in **Table 7**. Overall, screening was inconsistently performed: 40.8% of physicians reported screening children only sometimes, 26.1% rarely, and 4.1% never, while 29.0% reported screening most of the time or always. Similar patterns were observed for adolescents, with 43.3% reporting screening sometimes and 28.6% reporting screening most of the time or always. Screening was performed in adolescents (always or most of the time) by 40.3% of endocrinologists, followed by 32.6% of family medicine physicians and 19.3% of pediatricians. It was predominantly risk-based, most commonly prompted by obesity (89.4%), family history of dyslipidemia (86.5%), and family history of premature cardiovascular disease (86.5%). Universal screening was rarely reported as always performed (9% in children and 12.7% in adolescents). Among children with risk factors, additional metabolic testing was frequently prescribed, particularly fasting glucose, HbA1c, and TSH. The main perceived barriers to screening were the cost of the test (49.8%) and parental consent (33.9%), while lack of time (5.3%) and uncertainty about managing abnormal results (4.9%) were less frequently reported.

**Table 7:** Clinical practices for pediatric dyslipidemia screening.

Practices-related questions		Specialty			Total	p.value
		Pediatrics	Endocrinology	Family medicine		
How often do you screen for dyslipidemias in children in your practice?	Never	3 (2.5%)	4 (5.2%)	3 (6.1%)	10 (4.1%)	0.464
	Rarely	26 (21.8%)	23 (29.9%)	15 (30.6%)	64 (26.1%)	
	Sometimes	55 (46.2%)	28 (36.4%)	17 (34.7%)	100 (40.8%)	
	Most of the times	26 (21.8%)	16 (20.8%)	7 (14.3%)	49 (20.0%)	
	Always	9 (7.6%)	6 (7.8%)	7 (14.3%)	22 (9.0%)	
How often do you screen for dyslipidemias in adolescents in your practice?	Never	5 (4.2%)	1 (1.3%)	5 (10.2%)	11 (4.5%)	**0.011**
	Rarely	31 (26.1%)	14 (18.2%)	13 (26.5%)	58 (23.7%)	
	Sometimes	60 (50.4%)	31 (40.3%)	15 (30.6%)	106 (43.3%)	
	Most of the times	13 (10.9%)	15 (19.5%)	11 (22.4%)	39 (15.9%)	
	Always	10 (8.4%)	16 (20.8%)	5 (10.2%)	31 (12.7%)	
Which of these factors prompts you to screen for pediatric dyslipidemias?	Family history of a cardiovascular disease at a young age	103 (86.6%)	68 (88.3%)	41 (83.7%)	212 (86.5%)	0.758
	Family history of dyslipidemias	108 (90.8%)	64 (83.1%)	40 (81.6%)	212 (86.5%)	0.165
	Obesity	112 (94.1%)	64 (83.1%)	43 (87.8%)	219 (89.4%)	**0.047**
In the presence of risk factors (obesity, dyslipidemias in family, cardiovascular disease in family…) which other laboratory tests do you order?	Fasting Blood Glucose	94 (79.0%)	63 (81.8%)	38 (77.6%)	195 (79.6%)	0.824
	TSH	78 (65.5%)	67 (87.0%)	33 (67.3%)	178 (72.7%)	**0.003**
	HbA1c	101 (84.9%)	54 (70.1%)	30 (61.2%)	185 (75.5%)	0.002
	Insulin	62 (52.1%)	29 (37.7%)	14 (28.6%)	105 (42.9%)	**0.011**
What are the barriers to screening that you encounter most frequently?	Cost of the test	64 (53.8%)	30 (39.0%)	28 (57.1%)	122 (49.8%)	0.066
	Parent's consent	41 (34.5%)	25 (32.5%)	17 (34.7%)	83 (33.9%)	0.951
	Lack of time	8 (6.7%)	2 (2.6%)	3 (6.1%)	13 (5.3%)	0.435
Do not know what to do with abnormal results	No	114 (95.8%)	73 (94.8%)	46 (93.9%)	233 (95.1%)	0.862
	Yes	5 (4.2%)	4 (5.2%)	3 (6.1%)	12 (4.9%)	

Significant p values are shown in bold.

### Relationships between knowledge, attitudes, and practices

**Figure 1:**
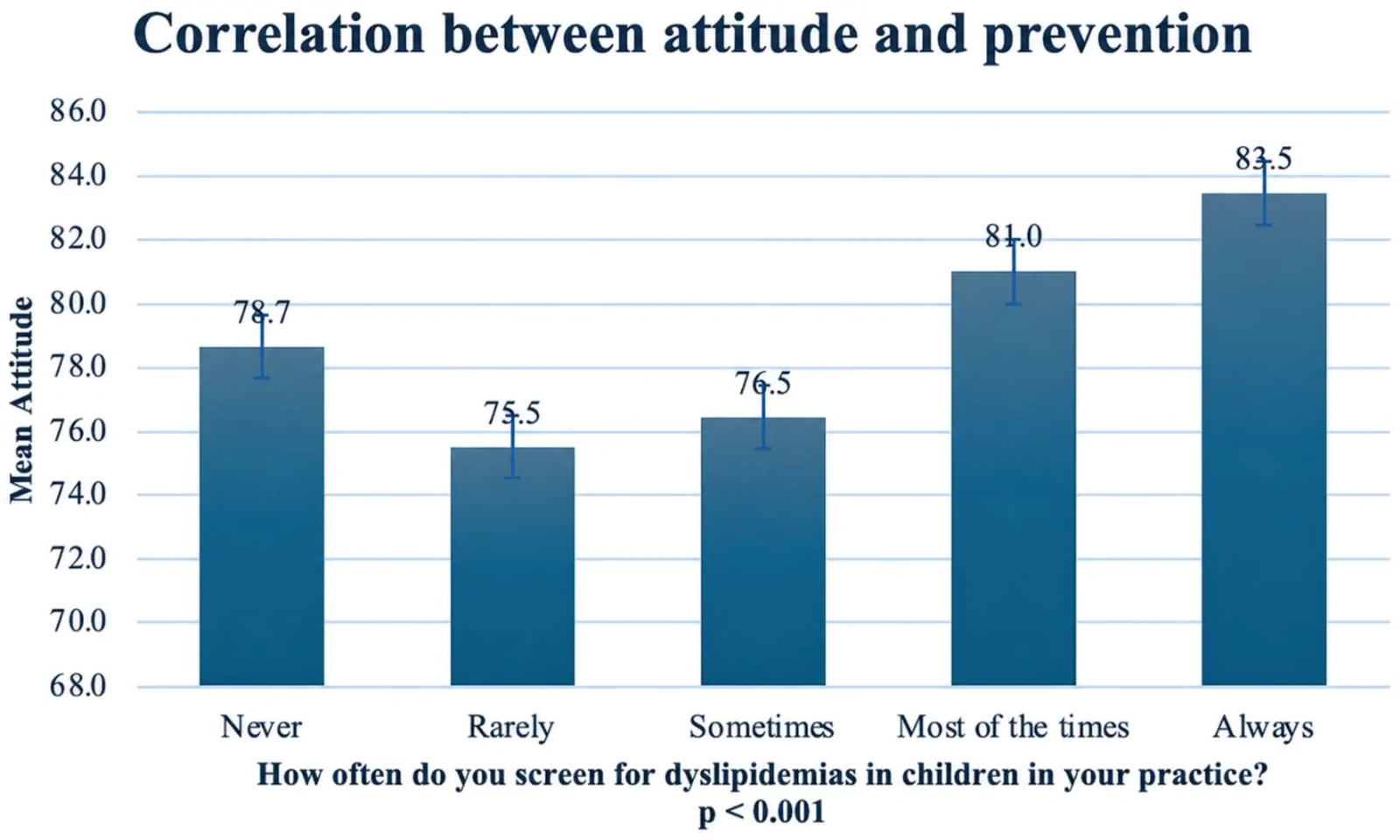
Mean attitude score according to reported screening frequency.

**Figure 2:**
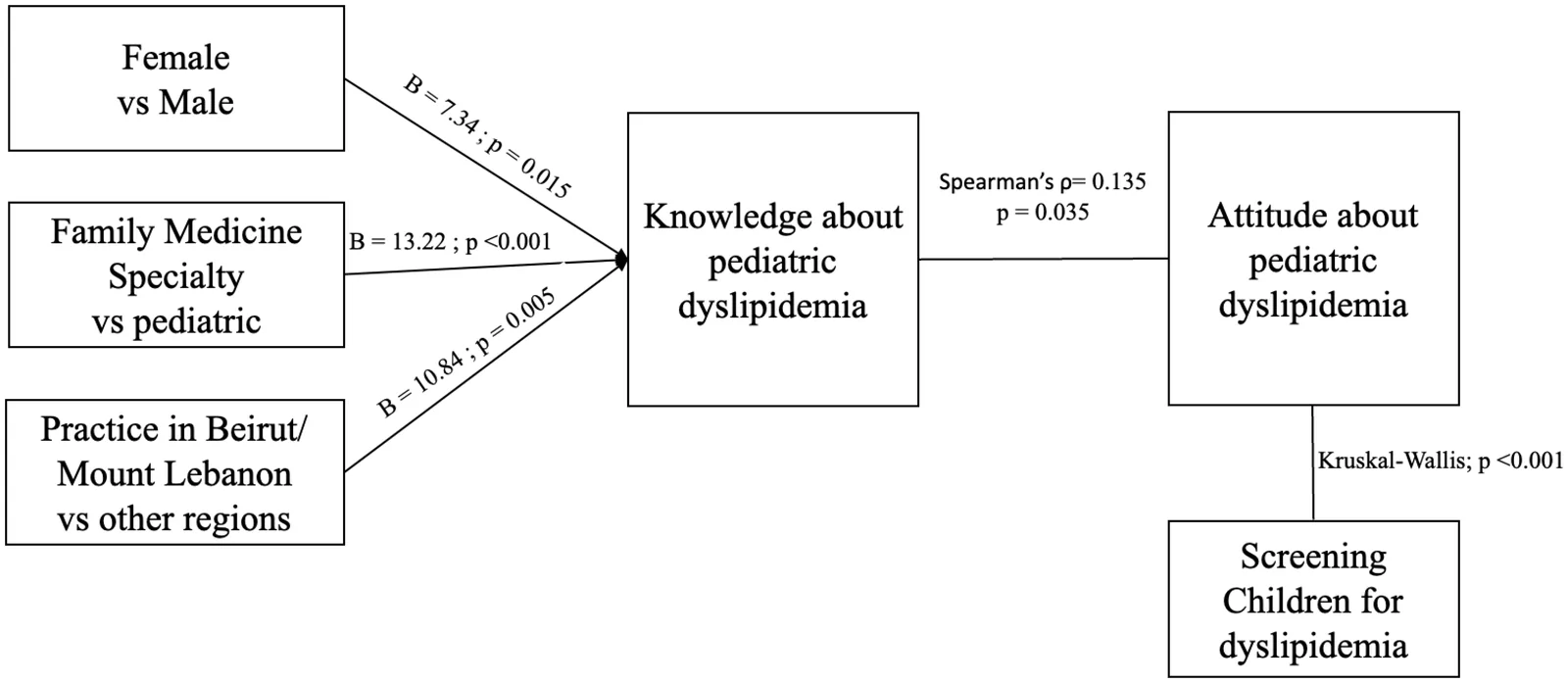
Summary of the main observed associations across the KAP domains.

Physicians who reported screening most of the time or always had higher mean attitude scores compared to those who never or rarely screened (83.5 and 81.0 vs. 75.5 and 78.7, respectively) (**Figure 1**). The score based on the nine selected knowledge items showed a weak positive correlation with attitude scores (Spearman’s ρ= 0.135, p = 0.035). Attitude scores differed significantly across screening frequency categories (Kruskal–Wallis H(4) = 20.20, p<0.001). **Figure 2** provides a descriptive summary of the main observed associations, presented as the KAP domains, and does not imply causal relationships.

## Discussion

To our knowledge, this is the first study conducted in Lebanon to jointly evaluate physicians’ knowledge, attitudes, and practices regarding pediatric dyslipidemia screening among pediatricians, family medicine physicians, and endocrinologists. It highlighted an insufficient level of knowledge among Lebanese surveyed physicians, as well as a substantial gap between favorable attitudes toward screening and its implementation in clinical practice. While the attitude scores reflected generally favorable perceptions toward screening, only 29% of physicians reported consistently (always or most often) applying these practices in children’s routine care.

The overall knowledge score among surveyed physicians remained insufficient (mean ± SD: 53.2 ± 22.1), particularly regarding estimates of the prevalence of pediatric dyslipidemias in Lebanon and familiarity with international recommendations. These findings are particularly concerning, given the role of dyslipidemia in the early development of atherosclerosis and cardiovascular diseases in adulthood (3, 4, 20, 21). This situation does not appear to be specific to the Lebanese context. Several North American studies have shown that, despite the availability of formal recommendations, physicians’ knowledge of and adherence to screening guidelines remain incomplete and vary across specialties and practice settings (17, 22, 23). Conflicting recommendations on universal screening between the NHLBI/AAP and USPSTF (8, 9, 11, 12) likely contribute to this variability and complicate the standardization of practices. The differences observed according to specialty are particularly interesting. In contrast to a previous study showing greater adherence to screening among pediatricians (10), our results indicate that family medicine physicians had higher scores on the nine selected knowledge items. This observation may reflect their central role in preventive care and the longitudinal follow-up of families. However, these aspects were not directly assessed in the present study, and differences in training, practice setting, or patient populations may also have contributed. Similarly, the higher scores observed among physicians practicing in BML may be related to greater access to continuing medical education, academic centers, or scientific resources. Nevertheless, access to these resources was not measured, and this association may also reflect other unmeasured regional characteristics. It should also be noted that many physicians whose main practice is located in BML may also provide care in other regions of the country on selected days of the week. This pattern may partly explain the high proportion of physicians classified as practicing in BML, as medical practice and specialist services in Lebanon are largely concentrated in this region. Finally, female physicians had higher scores based on the selected knowledge items. Although differences in exposure to continuing education or preventive-health information could be considered possible explanations, these mechanisms were not evaluated in our survey. This association should therefore not be interpreted as demonstrating greater engagement in preventive care among female physicians.

Despite these knowledge gaps, physicians’ attitudes toward screening were largely favorable. Most participants recognized pediatric dyslipidemia as an important public health issue and considered early screening an essential component of long-term cardiovascular prevention. This perception is consistent with evidence demonstrating the childhood origins of atherosclerotic risk and the cumulative effect of early exposure to elevated lipid levels (3, 4, 20, 21). However, these favorable attitudes were accompanied by important reservations, particularly concerning the potential anxiety caused to children and their families. Several factors may explain why these favorable attitudes did not translate into consistent practice. The ongoing scientific debate on the risk-benefit balance of universal screening, together with the absence of full agreement between professional societies, leaves physicians without a single clear standard to apply (9, 11, 12). Comparable observations have also been reported, where psychological, economic, and organizational considerations were shown to limit adherence to universal screening (10, 23). Notably, attitude scores in our sample were not associated with any sociodemographic characteristic, indicating that these perceptions were shared regardless of gender, years of experience, medical specialty, or region of practice, and therefore likely reflect uncertainties and constraints common to all participating physician groups.

Reported practices confirmed this discrepancy. Lipid screening remained predominantly risk-based, with family history and obesity being the most frequently reported indications, a pattern consistent with international studies documenting incomplete implementation of pediatric lipid screening recommendations (10, 17, 22, 23). Beyond physicians’ knowledge and attitudes, such practices appear to be related to contextual determinants specific to the setting in which care is delivered, including the absence of Lebanese-specific guidance, laboratory costs and unequal coverage, and the lack of standardized referral and follow-up pathways. Overall, only 9.0% and 12.7% of surveyed physicians reported always screening children and adolescents, respectively, with adolescent screening most frequently reported by endocrinologists, possibly reflecting specialty-specific practice patterns. Age-related patterns described in the literature have been inconsistent. A large U.S. study involving more than three million youths reported screening frequencies of 9% among those aged 9–11 years, 11.1% among those aged 12–16 years, and 12.9% among those aged 17–21 years (24). Although these proportions are numerically close to ours, the comparison should be made with caution, as that study measured patient-level screening documented in electronic medical records, whereas ours assessed physicians’ self-reported practices. In the study by de Ferranti et al. (17), approximately 32% of pediatricians regularly screened healthy 9-11-year-olds for cholesterol, and 42% screened 17–21-year-olds. The higher rates observed in adolescents may be due to the perception of greater cardiometabolic risk in this age group, or because lipid testing is often performed during transition-to-adult-care visits. Even among children attending routine care, however, screening completion remains limited, as only 33.3% of those aged 9–11 years seen for a well-child visit completed lipid screening (343/1,039) (25). The more cautious 2023 USPSTF position and COVID-19–related disruptions may have contributed to declining pediatric lipid screening and reinforced risk-based rather than universal approaches (5, 8, 9, 16, 17).

The barriers reported by our participants also differed from those described in several Western healthcare systems, where time constraints and clinical uncertainty predominate; in our setting, financial and organizational constraints played the central role. The cost of laboratory testing emerged as the main obstacle, reported by nearly 50% of physicians. In a country marked by a prolonged socioeconomic crisis and unequal healthcare coverage, this financial burden may constitute a major system-level barrier. The heterogeneity of the tests prescribed in the presence of risk factors further suggests the absence of harmonized national protocols, leading to variability in clinical practices. Indeed, no formally published national guideline, organized nationwide screening program, or unified professional-society recommendation specifically addressing pediatric dyslipidemia screening currently exists in Lebanon. Although the country has a broader National School Health Program and has resumed medical examinations for public school students, publicly available information does not indicate that systematic lipid testing is included.

Finally, our study examined the associations between physicians’ knowledge, attitudes, and practices regarding pediatric dyslipidemia screening. Knowledge scores were weakly associated with attitude scores, while attitude scores differed across reported screening-frequency categories. Given the cross-sectional design and the modest strength of these associations, these findings should not be interpreted as evidence of a causal pathway. They nevertheless suggest that factors beyond knowledge and attitudes may play an important role in clinical decision-making, particularly financial and organizational factors, such as screening costs and the lack of clear national guidelines. This interpretation is consistent with a recent study showing that implementation packages combining physician education, workflow integration, and collaboration with specialists were feasible and useful for improving FH screening in primary care (26). Similar implementation strategies may be relevant in Lebanon. Family medicine physicians, who achieved higher scores on the selected knowledge items, could play a key role in implementing and coordinating future screening initiatives.

Given the reported burden of lipid abnormalities among Lebanese youth, the progressive implementation of universal pediatric lipid screening could be considered an important national public health objective. The Ministry of Public Health, in collaboration with educational authorities and national pediatrics, endocrinology, family medicine, cardiology, and public health societies, could establish a multidisciplinary group to adapt international recommendations to the Lebanese context and define screening ages, testing methods, diagnostic thresholds, confirmatory procedures, and referral criteria. Implementation could begin through pilot programs involving primary healthcare centers, pediatric and family medicine practices, and existing school-health structures. These programs should be supported by physician training and parental education, particularly given the low levels of parental knowledge and reported screening practice observed in the Lebanese population (27). They should also include affordable or subsidized laboratory testing, standardized reporting procedures, and clearly defined pathways for lifestyle intervention, repeat testing, and specialist referral when FH is suspected. Screening uptake, detection rates, follow-up completion, feasibility, acceptability, cost-effectiveness, and potential harms should be evaluated before nationwide expansion.

This study has several strengths. First, it used an integrated approach based on the KAP model. Second, it included physicians from three key medical specialties and different geographic regions, allowing exploratory comparisons across participant subgroups. Third, it provided an in-depth analysis of factors associated with physicians’ knowledge and attitudes. In addition, although the sample size was modest, it was larger than that of the comparable U.S. study, which included 133 physicians (10). The overall response rate of approximately 39.6% among actively surveyed physicians also suggests a satisfactory level of participation for a voluntary physician survey.

However, several limitations should be considered. First, the nine screening-related knowledge items focused primarily on selected epidemiological findings, recommended screening ages and indications, pediatric lipid thresholds, and selected AAP/NHLBI recommendations. The nine selected knowledge items’ score should therefore be interpreted as performance on the selected items included in the questionnaire rather than as a comprehensive assessment of physicians’ overall knowledge of pediatric dyslipidemia. The items were not designed to evaluate in depth the comparative advantages, limitations, cost-effectiveness, and implementation requirements of universal screening, targeted risk-based screening, cascade screening for familial hypercholesterolemia, or school- or community-based screening strategies. In addition, the knowledge score demonstrated moderate internal consistency and limited sampling adequacy for factor analysis, which may partly reflect the heterogeneous content of the items. Second, the study used a voluntary, non-probability, society-based sampling strategy, and the sample was not designed to be nationally representative. Physicians practicing in BML and those affiliated with academic institutions were likely overrepresented, possibly reflecting the greater concentration of physicians and specialist services in BML. Furthermore, some specialty, geographic, and clinical-experience subgroups were relatively small, which may have reduced statistical power and the precision of subgroup estimates. Third, clinical practices were self-reported and were not independently verified using medical records or clinical audit data. Recall and social desirability biases may therefore have affected the reported screening frequencies. Participants may have reported practices perceived as professionally desirable or may not have accurately recalled their usual clinical practices. The practice findings should consequently be interpreted as self-reported practices rather than objectively measured clinical behavior. Finally, the cross-sectional design of the study does not allow temporal or causal relationships between knowledge, attitudes, and practices to be established. The observed associations require further confirmation through larger, nationally representative studies incorporating objective measures of clinical practice and more detailed assessments of training, access to educational resources, healthcare setting, and regional patterns of medical practice.

## Conclusion

To our knowledge, this is the first study conducted in Lebanon to jointly evaluate physicians’ knowledge, attitudes, and practices regarding pediatric dyslipidemia screening among pediatricians, family medicine physicians, and endocrinologists. Although physicians generally expressed favorable attitudes toward screening, performance on the selected knowledge items was insufficient, and self-reported screening practices were limited, highlighting a substantial gap between favorable attitudes and implementation. These findings suggest that improving physicians’ knowledge alone may not be sufficient to enhance screening practices. Addressing financial barriers, providing harmonized evidence-based recommendations, and strengthening physician education may be necessary to support more consistent pediatric lipid screening. Given the reported burden of lipid abnormalities among Lebanese youth, the feasibility, acceptability, and cost-effectiveness of progressively implementing a nationally coordinated pediatric lipid-screening strategy should be evaluated.

## Data Availability

The raw data supporting the conclusions of this article will be made available by the authors, without undue reservation.
