# Supplementary material for: Knowledge, attitudes, and practices of Lebanese physicians on the screening of pediatric dyslipidemias: a KAP cross-sectional study
Source: Front Endocrinol (Lausanne). 2026 Sep 9;17:1900463. doi: 10.3389/fendo.2026.1900463 (PMC13598830; doi:10.3389/fendo.2026.1900463)
Supplement: Supplementary file 1 [file DataSheet1.pdf]

Supplementary Table S1. Rationale, correct answers, and supporting references for the nine pediatric lipid-screening knowledge items

Item	Correct answer	Supporting reference	Rationale for inclusion
Estimated prevalence of familial hypercholesterolemia in Lebanon	1 in 100 or 1 in 200*	Ref. 6 and Supplementary Ref. S1	Assesses knowledge of the estimated prevalence of familial hypercholesterolemia in Lebanon
Estimated prevalence of elevated triglycerides among Lebanese children aged 8–18 years	~26%	Lebanese school-based study, Ref. 6;	Assesses knowledge of the estimated prevalence of hypertriglyceridemia
First recommended age window for universal lipid screening	9-11 years	NHLBI/AAP, Ref. 8	Assesses knowledge of the first universal screening window
Second recommended age window for universal lipid screening	17-21 years	NHLBI/AAP, Ref. 8	Assesses knowledge of the second universal screening window
Family history of premature cardiovascular disease as an indication for early screening	Correct	NHLBI/AAP, Ref. 8	Assesses recognition of family-history-based screening indications
Obesity as an indication for early screening	Correct	NHLBI/AAP, Ref. 8	Assesses recognition of obesity-related risk
Hypertension as an indication for early screening	Correct	NHLBI/AAP, Ref. 8	Assesses recognition of cardiovascular risk factors
Diabetes as an indication for early screening	Correct	NHLBI/AAP, Ref. 8	Assesses recognition of high-risk medical conditions
Which LDL-C and triglyceride values are considered high in children?	LDL-C ≥130 mg/dL and TG ≥100 mg/dL in children younger than 10 years	NHLBI/AAP, Ref. 8	Assesses knowledge of pediatric lipid interpretation and age-specific triglyceride thresholds

*Both 1 in 100 and 1 in 200 were accepted as correct answers.

Reference S1. Abifadel M, Rabès JP, Jambart S, et al. The molecular basis of familial hypercholesterolemia in Lebanon: spectrum of LDLR mutations and role of PCSK9 as a modifier gene. *Hum Mutat.* 2009;30(7):E682–E691.
Reference S2. Bell DA, Hooper AJ, Watts GF, Burnett JR. Mipomersen and other therapies for the treatment of severe familial hypercholesterolemia. Vasc Health Risk Manag. 2012;8:651-9.

NB The nine items were selected to assess three predefined areas: knowledge of Lebanese epidemiological findings, knowledge of guideline-recommended screening ages and indications, and knowledge of pediatric lipid thresholds. The four risk factors included in the multiple-response question were scored as separate knowledge items.
